# Supplementary material for: Factors to determine the adoption of online teaching in Tanzania’s Universities during the COVID-19 pandemic
Source: PLoS One. 2023 Oct 5;18(10):e0292065. doi: 10.1371/journal.pone.0292065 (PMC10553215; doi:10.1371/journal.pone.0292065)
Supplement: S1 Appendix — (DOCX) [file pone.0292065.s001.docx]

| **Appendix 1: Reliability Statistics for factors determine online courses teaching** | | | |
| --- | --- | --- | --- |
| **S/N** | **Context** | **Number of Items** | **Cronbach's Alpha** |
| 1 | **Knowledge & Technological factors** | It is very easy to prepare and deliver an online course | 88.9 |
|  |  | The ICT infrastructure in my university support online course(s) |  |
|  |  | The e-learning platform used by my University is of high quality |  |
|  |  | We have e-learning experts who support me to prepare and deliver the course (s) at my University |  |
|  |  | I have skills and knowledge of preparing and delivering online course |  |
|  |  | I have attended short training on how to prepare and deliver online courses |  |
|  |  | I have attended long training to deliver online courses |  |
|  |  | There is enough e-learning facilities and equipment such as computers and laptops and Internet facilities |  |
|  |  | There is stable Internet connection |  |
|  |  | The ICT tools are constantly upgraded to keep them current |  |
|  |  | Lecturers are trained and have relevant and appropriate skills on online learning |  |
|  |  | There is standby power-generating to facilitate online courses |  |
| 2 | **Organizational Factors** | Offering online courses will be very usefulness to University | 77.1 |
|  |  | We have enough Human Resources that is capable to prepare and deliver online courses at my University |  |
|  |  | The University has sufficient financial resources to finance preparation and delivery of online courses |  |
|  |  | Shifting to online course will benefit my University |  |
|  |  | Having online courses will have positive impact to our University |  |
|  |  | There is an enabling environment in place to support the use of online courses |  |
| 3 | **Environmental factors** | If the university start offering online courses will attract more students | 88.8 |
|  |  | If the university will offer online courses will defeat our competitors |  |
|  |  | If the university will prepare and deliver online courses, will attract more educational partners |  |
|  |  | The University popularity will increase if will offer online courses |  |
| 4 | **Nature of the course** | I cannot prepare and deliver online course (s) because the course content does not allow and support | 74.7 |
|  |  | I cannot prepare and deliver online course(s) because we do not have e-learning curriculum |  |
|  |  | *It need a lot of preparation to deliver online course(s) |  |
|  |  | *It need permission from Tanzania Commission of Universities to deliver online course(s) |  |
|  |  | *It need to changes the course syllabus to deliver online course(s) |  |
| 5 | **Staff attitudes towards online courses** | The universities in Tanzania should adopt the use of e-learning for teaching and learning | 84.2 |
|  |  | Online courses will facilitate and assist my overall teaching |  |
|  |  | Online courses will able me to plan better for my teaching |  |
|  |  | The academic staff has enough and relevant skills and knowledge to use online courses in teaching and learning |  |
|  |  | Online courses will help to overcome the problem of a shortage of learning resources. |  |
|  |  | Online courses will make education more effective |  |
|  |  | There is an adequate fund for the institution to acquire the necessary online teaching facilities |  |
|  |  | *Traditional courses contribute more to students’ learning than online courses. |  |
|  |  | *There is more difficult work involved to prepare online courses compared to traditional delivery of courses. |  |
|  |  | *The contribution of online courses to the college’s reputation is marginal. |  |
|  | Key: |  |  |
|  | *Item deleted |  |  |
